# Hip Hinge Kinematics and Movement Confidence Before and After Founder Exercise Instruction in Healthy Adults: A Preliminary Study

**DOI:** 10.3390/jfmk11030322

**Published:** 2026-08-20

**Authors:** William J. Hanney, Julia Chase, Tysen Coates, Christian Rodriguez-Rolas, Michael Massaracchio, Abigail W. Anderson

**Affiliations:** 1 Spine and Mobility (SAM) Lab, School of Kinesiology & Rehabilitation Sciences, University of Central Florida, Orlando, FL 32816, USA; jchasegym@gmail.com (J.C.); tysen.51.tc@gmail.com (T.C.); christianrodriguezrolas@yahoo.com (C.R.-R.); 2 Department of Physical Therapy, Long Island University, Brooklyn, NY 11201, USA; michael.masaracchio@liu.edu; 3 Rehabilitation and Modulation of Pain (RAMP) Lab, School of Kinesiology & Rehabilitation Sciences, University of Central Florida, Orlando, FL 32816, USA; abigail.wilson@ucf.edu

**Keywords:** founder exercise, hip hinge, movement instruction, kinematics, movement confidence, healthy adults

## Abstract

**Background**: Impaired lumbopelvic control and inefficient bending mechanics are associated with low back pain and functional limitations. The Founder Exercise is a movement retraining strategy intended to promote hip-dominant movement, trunk control, and postural awareness during forward bending. However, empirical evidence examining movement performance following Founder Exercise instruction is limited. This study examined immediate pre-to-post differences in hip hinge kinematics and movement confidence surrounding a standardized Founder Exercise instructional session in healthy adults. **Methods**: A within-subject pretest-posttest design was used. Thirty-three healthy adults (72.7% female; mean age, 25.1 ± 2.9 years) completed assessments of foot orientation, sagittal-plane joint kinematics, and movement confidence before and after a single Founder Exercise instructional session. Joint kinematics were assessed using two-dimensional video analysis. Paired-samples *t*-tests or Wilcoxon signed-rank tests, as appropriate based on the distributions of the paired differences, were used to evaluate the kinematic outcomes. The exploratory summed confidence score and individual ordinal confidence items were evaluated using Wilcoxon signed-rank tests. Holm adjustments were applied separately to the 11 kinematic comparisons and the 10 individual-item comparisons. **Results**: Statistically significant immediate pre-to-post differences were observed in several kinematic variables. Hip flexion increased from 80.7° ± 18.1° at pretest to 104.6° ± 14.3° at posttest, shoulder flexion increased from 62.8° ± 23.9° to 145.3° ± 13.9°, and craniovertebral angle decreased from 25.7° ± 12.9° to 11.2° ± 10.0° (all *p* < 0.001). The summed score from the unvalidated, study-specific confidence scale was higher at posttest (median = 49.0, IQR = 40.0–50.0) than at pretest (median = 40.0, IQR = 36.0–50.0; *p* < 0.001). After Holm adjustment of the individual ordinal-item analyses, statistically significant differences remained for five of the 10 items; all confidence findings were considered exploratory. No statistically significant differences were observed in knee flexion or ankle motion after Holm adjustment. **Conclusions**: A brief Founder Exercise instructional session was followed by immediate differences in selected two-dimensional hip hinge angles and higher exploratory movement-confidence scores in healthy adults. These findings do not establish changes in lumbopelvic control, muscle activation, spinal loading, movement efficiency, or clinical outcomes. Controlled studies incorporating direct measures of lumbar and pelvic motion, muscle activity, external forces, and clinically relevant outcomes are needed before biomechanical or clinical conclusions can be drawn.

## 1. Introduction

Low back pain is one of the leading causes of disability worldwide and represents a substantial burden on individuals, healthcare systems, and society [1]. Although low back pain is multifactorial in nature, impairments in lumbopelvic control and movement coordination during functional activities have been associated with both the development and persistence of symptoms [2]. Forward bending and lifting tasks are particularly relevant because they are commonly performed during daily activities and frequently provoke symptoms in individuals with low back pain. Efficient execution of these tasks requires appropriate coordination between the lumbar spine, pelvis, and hips in order to distribute mechanical demands across the kinetic chain [3,4].

The hip hinge is generally characterized by substantial hip motion during forward bending, although movement strategies vary across individuals and tasks. Hip-dominant movement is commonly emphasized during lifting and exercise; however, no isolated joint angle or universally accepted combination of angles defines an optimal hip hinge. Different lifting strategies may involve distinct mechanical advantages and disadvantages, and the mechanical consequences of a movement pattern cannot be determined from selected two-dimensional kinematic measures [2]. Accordingly, the present study examined whether selected joint positions changed immediately following instruction; it was not designed to determine whether the posttest pattern was biomechanically superior or clinically beneficial.

Hip-hinge strategies vary across populations and tasks. Altered lumbopelvic movement patterns have been associated with prospective functional decline in older adults with chronic low back pain [5], while deviations from neutral spinal posture have been observed during lifting exercises. These findings demonstrate variability in bending strategies but do not establish that a particular pattern is suboptimal, causes symptoms, or should be corrected. They provide context for investigating approaches to hip-hinge instruction but do not establish that changing isolated joint angles produces biomechanical or clinical benefits.

Instructional approaches to the hip hinge include using a dowel rod positioned along the spine to provide external feedback regarding spinal position. Although this approach provides a reproducible cueing method, the available evidence does not establish an optimal technique for teaching the hip hinge or demonstrate that any single instructed pattern produces superior biomechanical or clinical outcomes. Additional research is therefore needed to characterize how different instructional strategies affect measured task performance.

The Founder Exercise was developed as a movement-retraining strategy intended to promote posterior-chain engagement, trunk control, postural awareness, and hip-dominant movement during forward-bending activities. These represent proposed characteristics and mechanisms of the exercise rather than outcomes established by the present study. The exercise incorporates specific positioning of the feet, pelvis, trunk, upper extremities, and breathing mechanics while emphasizing active elongation of the spine and controlled hip flexion. Although the Founder Exercise is increasingly utilized within fitness and rehabilitation settings, empirical evidence examining movement performance following Founder Exercise instruction is limited.

The Founder Exercise warrants preliminary investigation as an instructional approach associated with hip hinge performance. Therefore, this study examined immediate pre-to-post differences in selected two-dimensional hip hinge kinematics and self-reported movement confidence surrounding a standardized Founder Exercise instructional session in healthy adults. Given the absence of a control or comparison condition, the study was designed to characterize temporal differences rather than isolate an intervention-specific effect, establish efficacy, or determine clinical benefit.

## 2. Materials and Methods

### 2.1. Study Design

This preliminary study used a single-group pretest–posttest design to examine immediate pre-to-post differences in selected two-dimensional hip hinge kinematics and self-reported movement confidence surrounding a standardized Founder Exercise instructional session in healthy adults. Pretest measurements reflected each participant’s existing understanding and performance of the hip hinge. Participants then completed standardized instruction and guided practice, after which the outcomes were reassessed immediately using a prompt that directed participants to apply their newly acquired knowledge and skill.

Because the study did not include a control or comparison condition, observed pre-to-post changes cannot be attributed specifically to the Founder Exercise. Practice, repeated testing, increased familiarity, expectancy, verbal or tactile cueing, and differences between the pretest and posttest instructions may have contributed to the findings. The study was not designed to evaluate treatment efficacy, clinical outcomes, or effectiveness in individuals with low back pain.

No criterion standard, composite movement-quality score, or predetermined angular threshold was used to classify an optimal hip hinge. Accordingly, statistically significant changes in individual kinematic variables were interpreted as immediate differences in task performance and, when applicable, as greater consistency with the movement characteristics emphasized during instruction. Changes in isolated joint angles were not classified as biomechanical improvements because their mechanical consequences cannot be determined without more comprehensive assessment of lumbar and pelvic motion, muscle activation, external forces, and spinal loading [2].

### 2.2. Participants

Participants were recruited from the University of Central Florida and the surrounding community. Inclusion criteria consisted of adults between 18 and 50 years of age who were able to perform physical activity and understand verbal and tactile instruction. Exclusion criteria included a current or prior history of significant low back, pelvic, or hip pain; musculoskeletal conditions affecting hip, knee, ankle, or foot function; neurological disorders affecting movement; and the presence of pain during testing or intervention procedures.

Healthy adults were intentionally selected to characterize immediate pre-to-post differences in movement performance surrounding standardized instruction without the potential influence of current pain, disability, fear of movement, or pain-related movement avoidance. The use of an asymptomatic sample was intended as a preliminary step before conducting controlled investigations in symptomatic populations. However, the present design cannot establish biomechanical effects or determine whether the observed changes would occur in individuals with low back pain.

### 2.3. Procedures

The study protocol consisted of three phases for each participant: (I) pretest assessment, (II) Founder Exercise instruction and guided practice, and (III) posttest assessment. Foot orientation, joint kinematics, and movement confidence were measured pretest and posttest, as outlined below.

#### 2.3.1. Foot Orientation

**Figure 1:**
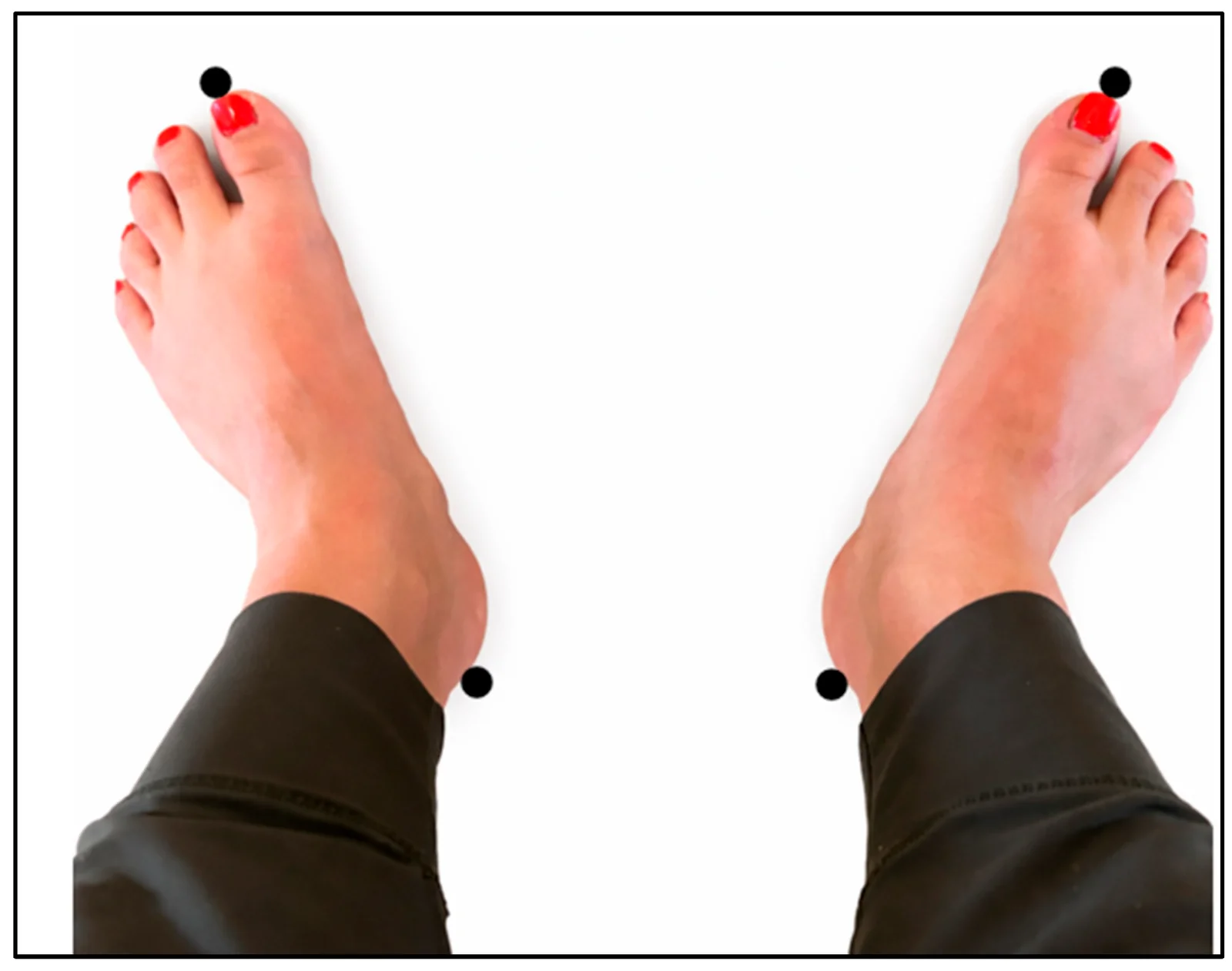
Setup used to measure foot orientation and stance width. The centers of the calcanei and distal ends of the great toes were marked to permit measurement of the distance between the calcanei and the orientation angle of each foot.

**Figure 2:**
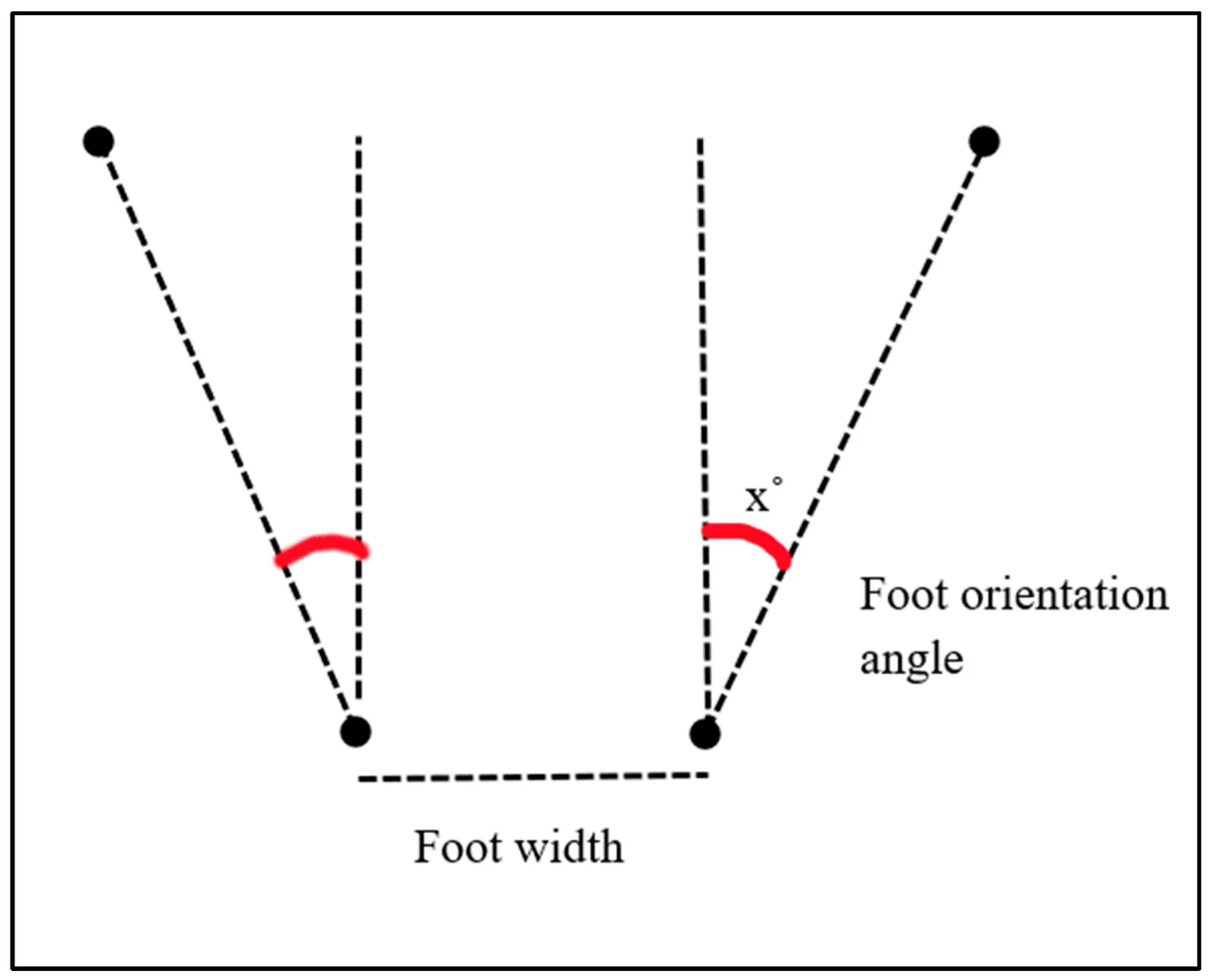
Measurement of the foot-orientation angle. The angle was measured between the line connecting the calcaneal center and distal great-toe point and the designated reference line.

The testing environment was prepared by rolling out a sheet of paper and securing it to the ground with tape. The participant was instructed to stand with both feet on the sheet of paper and adjust their feet to assume their desired starting position. Once set, the assessor marked the paper at the center of each calcaneus and at the distal end of each great toe. At the conclusion of the movement, the participant was instructed to step off the paper. The assessor measured the distance between the two center points with a tape measure and the angle between the center point and the great toe point of each foot using a goniometer. As described by McIlroy et al., the distance between the center of each calcaneus provides a measure of stance width, and the angle between the center point and great toe point provides the angle of orientation of the foot (see Figure 1 and Figure 2) [6].

#### 2.3.2. Joint Kinematics

Selected sagittal-plane joint angles were measured using two-dimensional video analysis with the OnForm application on an iPad. Two-dimensional video analysis can provide clinically accessible estimates of selected sagittal-plane kinematic variables, although it cannot fully characterize multiplanar movement or substitute for three-dimensional motion analysis [7]. Reflective markers were placed over the following anatomical landmarks on the left side of each participant: the C7 spinous process, mastoid process, lateral epicondyle of the humerus, humeral head, greater trochanter, lateral knee joint line, lateral malleolus, and base of the fifth metatarsal. Marker locations were verified by at least two members of the research team before recording.

The iPad was secured to a stationary tripod positioned approximately 3 m from the participant. The camera lens was placed 1.5 m above the floor and oriented perpendicular to the participant’s left sagittal plane, with the optical axis approximately level and centered on the participant’s hip. The camera was not moved between the pretest and posttest recordings. The participant’s entire body, including the upper extremities when elevated overhead, remained visible throughout each recording.

The recording frame rate and resolution were not documented at the time of data collection and therefore could not be confirmed retrospectively. No external spatial-calibration object was used because the analysis was limited to angular measurements constructed within OnForm from the marked anatomical landmarks; linear displacement measurements were not obtained.

During both the pretest and posttest assessments, participants completed three recorded hip-hinge repetitions following the corresponding standardized prompt. The middle repetition was selected for analysis. If that repetition could not be analyzed because of marker obstruction, loss of full-body visibility, or incomplete movement, the next chronologically recorded valid repetition was selected. Peak hinge depth was defined as the frame immediately before the participant began reversing from the descent phase to the ascent phase, corresponding to the greatest posterior displacement of the hips. If the peak position was maintained across multiple consecutive frames, the middle frame was selected; when an even number of frames showed the same peak position, the earlier of the two middle frames was used. Joint angles were measured in the OnForm application using the marked anatomical landmarks. The same examiner analyzed all recordings.

Inter-rater reliability and measurement error were evaluated using all 33 recordings, which were measured independently by two examiners. Agreement between examiners was estimated for ankle plantarflexion, ankle dorsiflexion, knee flexion, hip flexion, shoulder flexion, and craniovertebral angle using two-way random-effects, absolute-agreement, single-measure intraclass correlation coefficients [ICC(A,1)] with 95% confidence intervals [8]. The standard error of measurement (SEM) was calculated as SEM = SD_pooled_ × √(1 − ICC), and the minimal detectable change at the 95% confidence level (MDC95) was calculated as MDC95 = 1.96 × √2 × SEM.

Intra-rater reliability was evaluated using all 33 pretest recordings, which were reanalyzed by the same examiner after an interval of 48 to 72 h. The recordings were presented in randomized order during the second measurement session, and the examiner was blinded to the initial measurements. Repeatability was estimated for ankle plantarflexion, ankle dorsiflexion, knee flexion, hip flexion, shoulder flexion, and craniovertebral angle using two-way mixed-effects, absolute-agreement, single-measure intraclass correlation coefficients [ICC(A,1)] with 95% confidence intervals [8]. SEM and MDC95 were calculated using the formulas described above.

**Figure 3:**
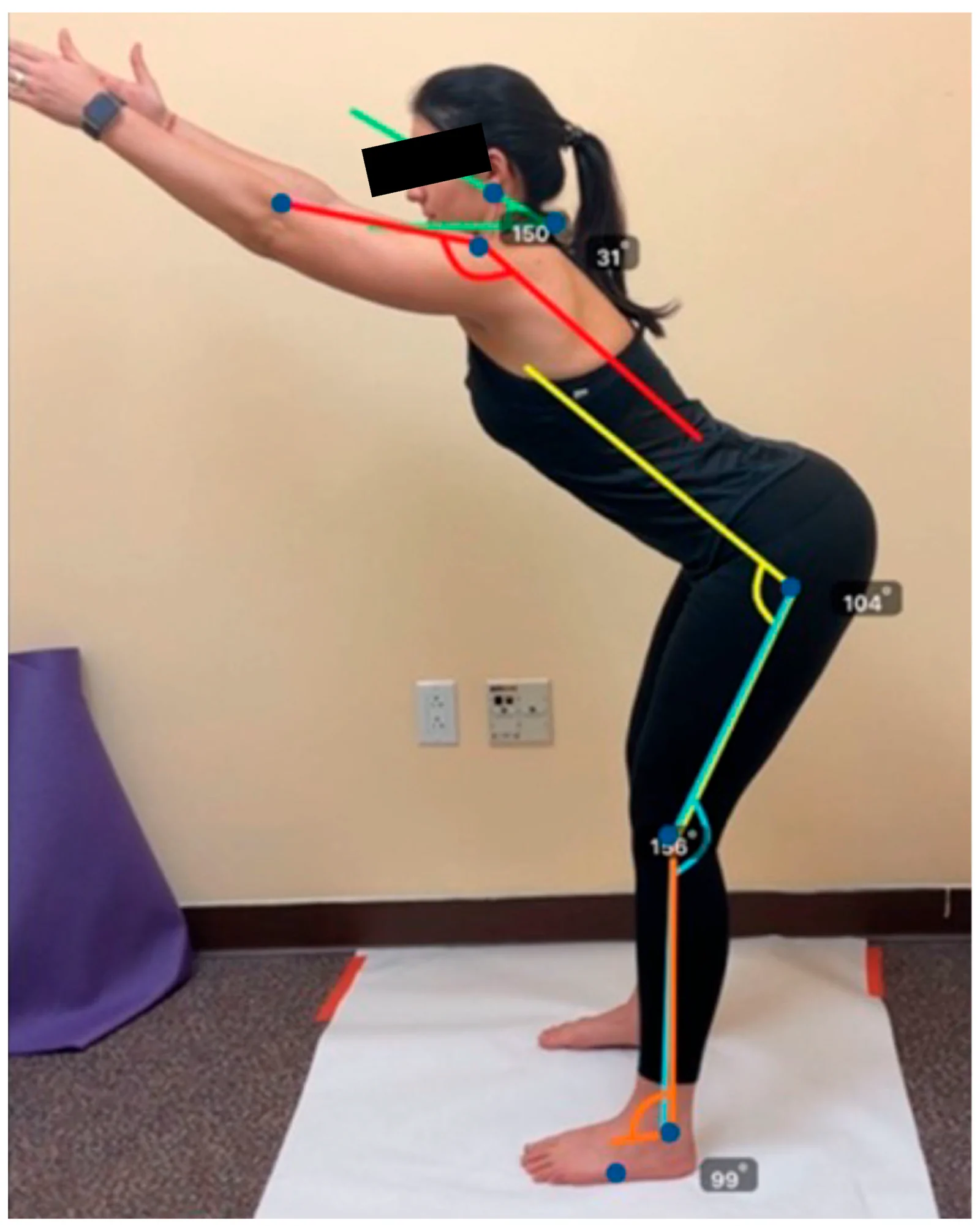
Representative pretest frame demonstrating two-dimensional joint-angle measurement using OnForm (v4.5.2). Blue dots identify the anatomical landmarks, and the colored lines represent the measurement segments used to calculate the craniovertebral, shoulder, hip, knee, and ankle angles. The numerical annotations are the raw angles displayed by OnForm for this representative participant and are not the group means reported in Table 2. Knee flexion was calculated as 180° minus the displayed knee included angle, and ankle motion was calculated as the angular deviation from the 90° reference.

**Figure 4:**
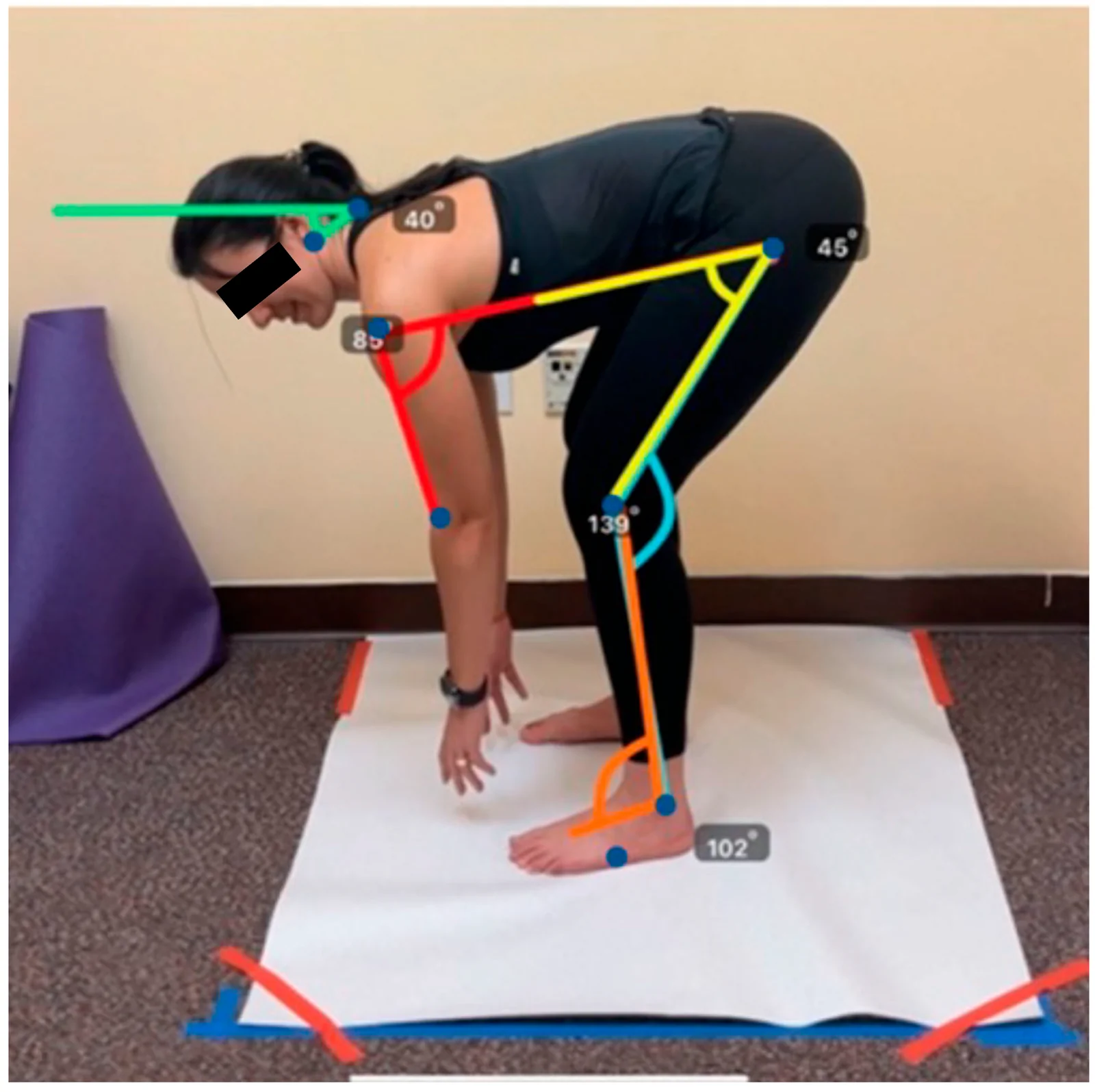
Representative posttest frame demonstrating two-dimensional joint-angle measurement using OnForm. Blue dots identify the anatomical landmarks, and the colored lines represent the measurement segments used to calculate the craniovertebral, shoulder, hip, knee, and ankle angles. The numerical annotations are the raw angles displayed by OnForm for this representative participant and are not the group means reported in Table 2. Knee flexion was calculated as 180° minus the displayed knee included angle, and ankle motion was calculated as the angular deviation from the 90° reference.

Craniovertebral angle was defined as the acute angle formed at C7 between a horizontal reference ray extending anteriorly from C7 and a second ray extending from C7 to the mastoid process. Under this measurement convention, a lower value indicated that the C7-to-mastoid line was more nearly parallel to the horizontal reference; a lower value was not independently classified as improved cervical alignment. Shoulder flexion was measured at the humeral head between a ray extending toward the lateral epicondyle of the humerus and a ray drawn parallel to the participant’s trunk. Hip flexion was measured at the greater trochanter between a ray extending toward the lateral knee joint line and a ray drawn parallel to the participant’s trunk. Knee position was displayed within OnForm as the included angle formed by the greater trochanter, lateral knee joint line, and lateral malleolus. For statistical analysis, knee flexion was calculated as 180° minus the displayed included angle. Ankle position was displayed as the included angle formed by the lateral knee joint line, lateral malleolus, and base of the fifth metatarsal. For statistical analysis, ankle motion was expressed as the angular deviation from the 90° reference and categorized as plantarflexion or dorsiflexion according to the direction of the deviation. Figure 3 and Figure 4 present representative pretest and posttest frames from one participant. The numerical annotations shown in these figures are the raw angles displayed by the OnForm application. As described above, the displayed knee and ankle included angles were transformed before statistical analysis. All videos were analyzed by the same examiner.

#### 2.3.3. Hip Hinge Confidence Scale

Participants completed the study-specific Hip Hinge Confidence Scale before and immediately after the instructional session. The scale contained 10 items assessing perceived confidence in performing a hip hinge during functional and exercise-related tasks, including lifting, bending, squatting, and lower-body exercise. Each item was rated on a five-point ordinal response scale ranging from 1 (“not at all confident”) to 5 (“extremely confident”), with higher scores indicating greater perceived confidence. Individual item scores were analyzed separately. An exploratory summed score was also calculated across the 10 items, producing a possible score ranging from 10 to 50. Because the scale was developed for this study and has not undergone formal psychometric validation, neither the individual items nor the summed score should be interpreted as validated participant-reported outcomes. Appendix A presents the complete scale.

Because no existing instrument was identified that specifically assessed confidence in performing hip hinge movements during functional activities, the questionnaire was developed specifically for this study. The scale has not undergone formal evaluation of its dimensionality, construct validity, test–retest reliability, or responsiveness. Internal consistency was estimated separately at pretest and posttest using Cronbach’s alpha. Internal consistency was high in this sample at pretest (α = 0.973) and posttest (α = 0.946). However, these sample-specific estimates do not constitute psychometric validation, demonstrate unidimensionality, or establish the validity, stability, or responsiveness of the scale. Accordingly, all individual-item and summed-score findings were treated as preliminary and exploratory.

The standardized Founder Exercise instructional session emphasized foot positioning, posterior displacement of the hips, trunk positioning, spinal elongation, upper-extremity positioning, and controlled breathing during forward bending. The intervention was delivered individually by the same instructor for all participants. At the time of the study, the instructor was a third-year Doctor of Physical Therapy student who had completed relevant training. Before participant enrollment, the instructor completed 4 h of training in the Founder Exercise. Intervention delivery was standardized using the scripted instructions and cueing procedures presented in Appendix B. The instructor also completed competency assessments and demonstrated adherence to the standardized protocol.

In Phase I, the pretest assessment began with the participant being instructed to perform a hip hinge to the best of their current ability. No practice repetitions were provided during this initial assessment in order to prevent an early learning effect. The assessor prompted the participant with, “Please perform a hip hinge movement based on your current understanding and ability.” An assessor collected all initial measurements as outlined above.

During Phase II, each participant received approximately 8 min of individual instruction from the designated instructor on how to perform the Founder Exercise using the standardized instructions and cueing procedures presented in Appendix B. Participants then completed 10 practice repetitions while receiving the standardized verbal and tactile cues described in Appendix B. The same instructor delivered the intervention to every participant. The instructor was different from the examiner who performed the kinematic measurements; however, the examiner was not blinded to assessment order.

Finally, in Phase III, the posttest assessment, the participant was prompted with, “With your new knowledge and skill, please perform a final hip hinge movement.” All posttest measurements were completed by the same examiner who performed the pretest measurements to reduce between-examiner variability.

### 2.4. Statistical Analysis

Statistical analyses were performed using JASP (version 0.16.1; JASP Team, Amsterdam, The Netherlands). Participant characteristics were summarized using means and standard deviations for continuous variables and frequencies and percentages for categorical variables. Study outcomes were summarized using means and standard deviations or medians and interquartile ranges, as appropriate for the corresponding analysis. All statistical tests were two-sided, with the family wise significance level established at α = 0.05.

No outcomes were prospectively designated as primary or secondary. Given the preliminary nature of the study, all pre-to-post kinematic and confidence outcomes were treated as exploratory. The sample consisted of 33 participants who completed the study. No formal a priori sample-size calculation was performed; the sample size was based on feasibility and the number of eligible participants recruited during the study period. Accordingly, the study was not powered to establish intervention efficacy or confirm effects for any individual outcome. All statistical findings should therefore be interpreted as exploratory and hypothesis-generating.

Paired-samples *t*-tests or Wilcoxon signed-rank tests, as appropriate based on the distribution of the paired differences, were used to evaluate immediate pre-to-post differences in the kinematic outcomes. Because the individual confidence items were measured on a five-point ordinal scale and exhibited restricted response distributions, pre-to-post differences in the individual items were evaluated using Wilcoxon signed-rank tests. For each continuous outcome, the normality assumption was evaluated using the distribution of the paired difference scores through visual inspection of histograms and quantile–quantile plots and the Shapiro–Wilk test. Potential extreme outliers were examined using boxplots and verified against the original data records. Paired-samples *t*-tests were used when the paired differences were approximately normally distributed and contained no unresolved extreme outliers; otherwise, Wilcoxon signed-rank tests were used. Independence was addressed through the study design because each participant contributed one paired observation and participants were analyzed independently. Wilcoxon signed-rank results for the individual confidence items were reported using the test statistic, unadjusted and Holm-adjusted *p*-values, and matched-pairs rank-biserial correlation as an effect-size estimate.

One participant had a missing pretest value for left-foot external rotation; therefore, this outcome was analyzed using 32 complete pairs. All other outcomes had complete data and were analyzed using all 33 participants. No observations were excluded as outliers. If values were missing, analyses were performed using available paired observations without imputation, and the number included in each analysis was reported. Mean differences were calculated as pretest minus posttest and reported with 95% confidence intervals. Standardized effect sizes for paired comparisons were calculated as Cohen’s *d_z_* by dividing the mean paired difference by the standard deviation of the paired differences. Under this convention, a negative *d_z_* indicates that the posttest mean was higher than the pretest mean, whereas a positive *d_z_* indicates that the posttest mean was lower than the pretest mean. Effect-size magnitude was interpreted using the absolute value of *d_z_*.

The summed confidence score was calculated only for participants who completed all 10 items. The exploratory 10-item summed confidence score was analyzed separately using a Wilcoxon signed-rank test because its paired differences were not normally distributed. Pretest and posttest values were summarized using medians and interquartile ranges, and the analysis included the Wilcoxon signed-rank statistic, unadjusted *p*-value, and matched-pairs rank-biserial correlation. Internal consistency of the 10-item scale was estimated separately at pretest and posttest using Cronbach’s alpha. Because the scale was developed specifically for this study and has not undergone formal psychometric validation, the summed-score and internal-consistency analyses were considered exploratory.

Statistical significance and standardized effect size were not interpreted as evidence of clinical importance. Observed mean differences were considered in relation to outcome-specific SEM and MDC95 estimates when verified reliability data were available. MDC95 was used only to describe whether an individual change was likely to exceed the measurement error; it was not interpreted as a minimal clinically important difference. No established minimal clinically important difference values were identified for the two-dimensional hip hinge angles that were assessed in healthy adults. Because the study did not measure pain, disability, physical function, task performance, or another validated clinical outcome, clinical meaningfulness could not be determined.

Because multiple exploratory comparisons were performed, the familywise type I error rate was controlled using the sequential Holm procedure. The procedure was applied separately to the 11 kinematic comparisons and the 10 individual confidence-item comparisons because these represented conceptually distinct outcome domains. The analysis of the exploratory summed confidence score was reported separately because it represented a single composite analysis rather than an additional individual-item comparison. Both unadjusted and Holm-adjusted *p*-values are reported for the kinematic and individual-item analyses, and statistical conclusions are based on the adjusted values. Because the outcome families and primary outcomes were not prospectively specified, effect estimates and 95% confidence intervals are emphasized, and all findings are interpreted as exploratory.

## 3. Results

### 3.1. Participant Demographics 

The study included 33 participants (72.7% female) with a mean age of 25.09 years (SD = 2.85). Mean self-reported height and weight were 168.89 cm (SD = 9.28) and 73.57 kg (SD = 19.82), respectively. Participant characteristics are summarized in Table 1.

The normality assumption was evaluated using the paired difference scores. Shapiro–Wilk tests did not indicate departures from normality for stance width, left-foot internal rotation, right-foot internal rotation, ankle plantarflexion, hip flexion, shoulder flexion, or craniovertebral angle (all *p* ≥ 0.135). Difference scores were not normally distributed for left-foot external rotation, right-foot external rotation, ankle dorsiflexion, and knee flexion (all *p* < 0.001); these outcomes were evaluated using Wilcoxon signed-rank tests. The summed confidence-score differences were also nonnormally distributed (*W* = 0.886, *p* = 0.002) and were evaluated using a Wilcoxon signed-rank test.

### 3.2. Joint Kinematics Results

Immediate pre-to-post differences in the kinematic outcomes are reported in Table 2. After Holm adjustment across the 11 kinematic comparisons, statistically significant differences remained for stance width, left-foot external rotation, left-foot internal rotation, right-foot external rotation, right-foot internal rotation, hip flexion, shoulder flexion, and craniovertebral angle. Ankle plantarflexion, ankle dorsiflexion, and knee flexion did not differ significantly after adjustment. Complete results are presented in Table 2.

### 3.3. Inter-Rater Reliability and Measurement Error

Inter-rater reliability and measurement-error estimates for the two-dimensional kinematic measurements are presented in Table 3. ICC(A,1) estimates ranged from 0.534 to 0.998. Five measurements demonstrated excellent agreement (ICC = 0.978–0.998), whereas ankle dorsiflexion demonstrated moderate agreement (ICC = 0.534; 95% CI, −0.058 to 0.742). SEM values ranged from 0.72° to 1.86°, and MDC95 values ranged from 1.99° to 5.14°. Confidence intervals were relatively narrow for the five measurements, demonstrating excellent agreement, but wide for ankle dorsiflexion, indicating substantial uncertainty in that estimate.

### 3.4. Intra-Rater Reliability and Measurement Error

Intra-rater reliability and measurement-error estimates are presented in Table 4. ICC(A,1) estimates ranged from 0.534 to 1.000. Ankle plantarflexion, knee flexion, hip flexion, shoulder flexion, and craniovertebral angle demonstrated excellent intra-rater reliability (ICC = 0.985–1.000). Ankle dorsiflexion demonstrated moderate reliability (ICC = 0.534; 95% CI, 0.235–0.740). SEM values ranged from 0.51° to 0.95°, and MDC95 values ranged from 1.41° to 2.64°.

### 3.5. Summed Hip Hinge Confidence Scale Score

In a separate exploratory composite analysis, the summed confidence score was higher at posttest (median = 49.0, IQR = 40.0–50.0) than at pretest (median = 40.0, IQR = 36.0–50.0). Because the paired differences were not normally distributed, a Wilcoxon signed-rank test was used and demonstrated a statistically significant difference (*W* = 22.5, *p* < 0.001; matched-pairs rank-biserial correlation = 0.822). This composite analysis was not included in the Holm adjustment applied to the 10 individual-item comparisons and should be interpreted as exploratory. Internal consistency was high in this sample at pretest (α = 0.973) and posttest (α = 0.946). These sample-specific internal-consistency estimates do not establish the dimensionality, validity, test–retest reliability, or responsiveness of the scale. Accordingly, the individual-item and summed-score findings should be strictly interpreted as preliminary and exploratory.

Individual-item results are presented in Table 5. Wilcoxon signed-rank tests indicated higher posttest responses across the 10 items. After Holm adjustment, statistically significant differences remained for Items 1, 3, 5, 7, and 10. Items 2, 4, 6, 8, and 9 did not meet the adjusted significance threshold. Pretest ceiling effects were evident: between 36.4% and 51.5% of participants selected the highest response option across the individual items. These restricted baseline distributions may have limited the scale’s ability to detect higher posttest responses.

Because confidence was assessed immediately after instruction and the posttest prompt emphasized participants’ newly acquired knowledge and skill, the findings may have been influenced by task familiarity, expectancy, demand characteristics, social desirability, or participants’ awareness of the anticipated response. The results therefore cannot establish a durable change in movement confidence.

## 4. Discussion

This preliminary study examined immediate pre-to-post differences in selected two-dimensional hip-hinge kinematics and self-reported movement confidence surrounding a standardized Founder Exercise instructional session in healthy adults. After Holm adjustment, statistically significant differences were observed in hip flexion, shoulder flexion, craniovertebral angle, selected foot-orientation outcomes, and five of the 10 individual confidence items; the exploratory summed confidence score was also higher at posttest. These findings indicate that participants performed the assessed task differently and reported higher confidence scores at the immediate posttest. However, because the design did not include a control or comparison condition, the observed differences cannot be attributed specifically to the Founder Exercise and may reflect practice, repeated testing, increased task familiarity, expectancy, differences in the assessment prompts, Hawthorne effects, or assessor cueing. The findings also do not establish that the posttest movement represented a biomechanical or clinical improvement.

At posttest, participants demonstrated greater hip and shoulder flexion, a lower craniovertebral angle, and differences in foot positioning. These findings indicate that selected two-dimensional joint angles differed immediately after instruction and that several of these differences were consistent with the movement positions emphasized during the instructional session. They do not demonstrate improved movement quality or establish changes in lumbopelvic control, posterior-chain muscle activation, spinal loading, movement efficiency, or injury risk because these variables were not directly measured. Furthermore, no single lifting strategy has been established as universally biomechanically superior, and different movement strategies may involve distinct mechanical advantages and disadvantages [2]. Any proposed neuromuscular or mechanical consequences of the observed angular differences should therefore be regarded as hypotheses requiring direct evaluation.

Exercise, movement-control training, and individualized movement instruction are incorporated into contemporary physical therapy management for some individuals with low back pain [9]. However, the present sample consisted exclusively of young, healthy, asymptomatic adults, and the study did not assess pain, disability, function, recurrence, or other clinical outcomes. Additionally, the evidence does not support prescribing one lifting technique as universally preferable for preventing or managing low back pain [2,5]. Accordingly, the present findings provide no evidence that Founder Exercise instruction is effective for treating or preventing low back pain. Instead, they provide preliminary descriptive information that may inform the design of future controlled studies involving symptomatic populations.

Exploratory analyses indicated that the summed movement-confidence score was higher immediately after instruction. Individual-item analyses were less uniform: after accounting for the ordinal response format and applying the Holm procedure, five of the 10 items demonstrated statistically significant pre-to-post differences. Confidence and self-efficacy may influence movement participation and functional behavior, and greater self-efficacy has been associated with more favorable outcomes among individuals with chronic musculoskeletal pain [10]. However, the study-specific construct assessed here was task-specific hip hinge confidence in healthy adults and should not be considered equivalent to validated pain self-efficacy.

Interpretation is also limited by the high baseline scores and restricted response range. Between 36.4% and 51.5% of participants selected the maximum response at pretest across the individual items, indicating potential ceiling effects and limiting the opportunity to observe higher posttest responses. Moreover, confidence was reassessed immediately after participants received instruction and practiced the same movement represented in the questionnaire. The posttest prompt explicitly emphasized their newly acquired knowledge and skill. Consequently, the observed responses may have been influenced by task familiarity, expectancy, demand characteristics, social desirability, or awareness of the study purpose. These preliminary findings should not be interpreted as evidence of a durable change in confidence or as evidence supporting the validity or responsiveness of the study-specific scale.

Verbal, visual, and tactile feedback can produce immediate changes in motor-task performance, particularly when participants receive feedback while practicing the assessed movement. However, improved performance immediately following practice should be distinguished from motor learning, which is more appropriately evaluated through delayed retention or transfer testing after the temporary effects of practice and feedback have diminished [11]. Because the present study did not include a control condition, it cannot determine whether the observed changes were specific to Founder Exercise instruction or whether similar changes would occur following conventional hip hinge instruction, guided practice, repeated testing, or another feedback-based approach. Controlled comparative studies incorporating retention and transfer assessments are needed to determine whether differences associated with Founder Exercise instruction exceed those associated with task familiarization, repeated testing, conventional hip hinge instruction, guided practice, or another feedback-based approach and whether any differences persist after feedback is withdrawn.

Pre-to-post differences were observed in hip, shoulder, and craniovertebral angles, whereas knee and ankle angles did not differ significantly after adjustment for multiple comparisons. This pattern identifies where differences occurred among the measured variables but does not explain the neuromuscular or mechanical mechanisms underlying them. Although a hip hinge is commonly described as involving substantial hip contribution, selected two-dimensional joint angles cannot quantify lumbar–pelvic coordination, muscle recruitment, external forces, tissue loading, or movement efficiency. Greater posterior-chain activation, improved proximal control, and altered spinal loading may be examined as hypotheses in future studies using electromyography, three-dimensional motion analysis, and kinetic measurements; they are not findings of the present study.

The observed mean pre-to-post differences in ankle plantarflexion, hip flexion, shoulder flexion, and craniovertebral angle exceeded their respective MDC95 values, whereas the differences in ankle dorsiflexion and knee flexion did not. These comparisons should be interpreted cautiously because MDC95 estimates the magnitude of change required to exceed the measurement error for an individual measurement; it is not a minimal clinically important difference or a test of group-level statistical significance. Exceeding MDC95 therefore does not establish clinical importance, biomechanical superiority, or an effect attributable specifically to the instructional session.

Several limitations should be considered when interpreting these findings. Most importantly, the single-group pretest–posttest design lacked a control or comparison condition. Consequently, the observed differences cannot be attributed specifically to the Founder Exercise or to learning and practice effects, repeated testing, increased task familiarity, expectancy, Hawthorne effects, assessor cueing, or the immediate influence of verbal and tactile feedback. In addition, the pretest prompt asked participants to perform the movement according to their existing understanding, whereas the posttest prompt directed them to apply their newly acquired knowledge and skill. This difference in assessment instructions represents an additional potential source of performance and response bias. Accordingly, the findings demonstrate temporal associations only and do not support causal conclusions regarding the Founder Exercise.

The sample was relatively small and homogeneous and consisted primarily of young, healthy adults. No a priori sample-size calculation was performed, and the sample was determined pragmatically. Because no primary outcome, prespecified clinically meaningful difference, or validated clinical threshold was identified prospectively, the study cannot be considered adequately powered to detect clinically meaningful differences. The absence of statistically significant findings for some outcomes may therefore reflect insufficient statistical sensitivity rather than the absence of a meaningful difference. Conversely, statistically significant differences should not automatically be interpreted as clinically meaningful, particularly because the sample was asymptomatic and several outcomes do not have established minimal clinically important difference values for this context. Consequently, the findings should not be generalized to older adults, individuals with musculoskeletal disorders, or patients with low back pain.

Outcomes were only assessed immediately after instruction. Immediate changes in task performance do not necessarily represent durable motor learning, which is more appropriately evaluated using delayed retention or transfer testing [11]. Therefore, the persistence and transferability of the observed changes remain unknown. Kinematic measurements were obtained using two-dimensional video analysis of selected joint angles. This approach cannot fully characterize multiplanar movement, segmental lumbar–pelvic coordination, muscle activation, external forces, spinal loading, or movement efficiency. Consequently, the present findings should not be interpreted as evidence of improved neuromuscular control, posterior-chain recruitment, spinal mechanics, movement quality, or mechanical efficiency. These remain untested hypotheses that require direct evaluation using three-dimensional kinematics, electromyography, and kinetic measurements.

Movement confidence was assessed using a study-specific questionnaire that has not undergone formal psychometric validation. Although internal consistency was high in this sample, Cronbach’s alpha alone does not establish unidimensionality, construct validity, test–retest reliability, or responsiveness [12]. The confidence findings therefore represent exploratory responses to the study-specific items rather than validated participant-reported outcomes. Several items exhibited substantial pretest ceiling effects, which restricted the available range for observing higher posttest responses. In addition, immediate post-instruction responses may have been influenced by expectancy, demand characteristics, social desirability, task familiarity, or participants’ awareness of the study purpose.

Inter-rater and intra-rater reliability and measurement error were evaluated using all 33 recordings. Five measurements demonstrated excellent inter-rater and intra-rater agreement. Ankle dorsiflexion demonstrated only moderate agreement in both analyses and should therefore be interpreted cautiously. The intra-rater analysis was limited to repeated measurements of the pretest recordings and may not fully characterize measurement repeatability across all assessment conditions. In addition, MDC95 represents measurement error at the individual level and does not establish that the observed group differences were clinically meaningful. Future controlled studies should confirm these reliability estimates across additional examiners, assessment sessions, and clinically relevant populations; use validated clinical outcome measures; incorporate three-dimensional motion analysis; and include delayed retention and transfer assessments.

Multiple comparisons were conducted across the kinematic and confidence-related outcomes. The Holm procedure was applied separately to the 11 kinematic comparisons and the 10 individual confidence-item comparisons to control the familywise type I error rate within each outcome domain. Nevertheless, the analyses were exploratory, the outcome families were not prospectively specified, and no outcome was a priori designated as the primary outcome. The separate summed-confidence analysis was not included in either adjustment family. Accordingly, the possibility of false-positive findings cannot be eliminated, and the results require confirmation in adequately powered studies with prospectively defined primary outcomes and analysis plans.

Clinical relevance could not be determined in this study. The participants were healthy and asymptomatic, and the study did not assess pain, disability, physical function, lifting performance, patient-perceived improvement, injury risk, or participation. No established MCID values were available for the task-specific two-dimensional joint angles or the unvalidated confidence scale. In addition, effect-size magnitude describes the size of a standardized difference but does not establish that the difference is clinically meaningful. Consequently, the findings should not be interpreted as evidence that Founder Exercise instruction reduces pain, prevents injury, improves function, or produces a meaningful clinical benefit. Contemporary clinical guidelines support exercise and movement-control interventions for selected individuals with low back pain, but such recommendations are based on studies directly evaluating symptomatic populations and clinically relevant outcomes [9]. The present study identified immediate differences in selected kinematic measures and self-reported movement confidence following instruction; it did not establish that these differences represented superior movement quality or clinically meaningful change. Future controlled studies should directly evaluate individuals with low back pain and incorporate validated measures of pain, disability, function, pain self-efficacy, and movement confidence. Delayed retention and transfer assessments should also be included to determine whether observed changes persist beyond the immediate effects of instruction and feedback.

Future investigations should examine whether the observed differences are retained over time and compare Founder Exercise instruction with other instructional approaches, such as dowel-rod training. Studies incorporating larger and more diverse samples, three-dimensional motion analysis, and prospective clinical outcomes are also needed to determine whether Founder Exercise instruction has applications within rehabilitation or performance settings.

This exploratory single-group study found immediate pretest-to-posttest differences in selected two-dimensional joint angles and self-reported movement confidence surrounding a brief Founder Exercise instructional session. Because the design did not include a control condition, the observed differences cannot be attributed specifically to the instruction and may reflect repetition, testing, cueing, or other contextual effects. The findings also do not establish biomechanical superiority, functional improvement, clinical efficacy, or durable change. Because the sample consisted primarily of young, healthy adults, these findings should not be generalized to older adults, individuals with low back pain or other musculoskeletal conditions, people with movement impairments, or athletic populations. Controlled studies involving more diverse and clinically relevant samples are needed to determine whether these findings are reproducible, retained over time, transferable to functional tasks, and associated with meaningful clinical outcomes.

## Figures and Tables

**Table 1:** Participant demographic and anthropometric characteristics (*n* = 33).

Female	24 (72.7%)
Male	9 (27.3%)
Age (years)	25.091 ± 2.854
Height (cm)	168.890 ± 9.276
Weight (kg)	73.567 ± 19.819

Note. Data are presented as mean ± standard deviation unless otherwise indicated. *n* = number of participants; % = percentage of participants.

**Table 2:** Exploratory pretest-to-posttest differences in joint kinematic measures.

**Outcome**	***n***	**Pretest, Mean (SD) or Median (IQR)**	**Posttest, Mean (SD) or Median (IQR)**	**Change Estimate (95% CI)**	**Test Statistic**	**Unadjusted *p***	**Holm-Adjusted *p***	**Effect Size**
Stance width (cm)	33	29.1 (4.7)	34.0 (5.6)	−4.9 (−6.3 to −3.4)	t(32) = −6.782	<0.001	<0.001	*d_z_* = −1.181
Left-foot external rotation (°)	32	0.0 (0.0–1.0)	0.0 (0.0–0.0)	0.5 (0.0 to 1.0)	W = 0.0	<0.001	0.003	*r_rb_* = 1.000
Left-foot internal rotation (°)	33	2.3 (3.1)	11.6 (5.0)	−9.3 (−11.0 to −7.5)	t(32) = −10.690	<0.001	<0.001	*d_z_* = −1.861
Right-foot external rotation (°)	33	1.0 (0.0–5.0)	0.0 (0.0–0.0)	2.0 (0.5 to 3.0)	W = 4.0	<0.001	<0.001	*r_rb_* = 0.962
Right-foot internal rotation (°)	33	1.1 (1.8)	10.1 (6.0)	−9.0 (−10.9 to −7.1)	t(32) = −9.638	<0.001	<0.001	*d_z_* = −1.678
Ankle plantarflexion (°)	33	12.0 (5.9)	9.7 (6.2)	2.3 (−0.1 to 4.8)	t(32) = 1.915	0.064	0.193	*d_z_* = 0.333
Ankle dorsiflexion (°)	33	0.0 (0.0–0.0)	0.0 (0.0–0.0)	0.0 (0.0 to 0.0)	W = 1.5	0.414	0.462	*r_rb_* = 0.500
Knee flexion (°)	33	27.0 (18.0–34.0)	24.0 (18.0–35.0)	3.5 (−2.0 to 9.0)	W = 213.5	0.231	0.462	*r_rb_* = 0.239
Hip flexion (°)	33	80.7 (18.1)	104.6 (14.3)	−23.9 (−29.9 to −17.8)	t(32) = −8.052	<0.001	<0.001	*d_z_* = −1.402
Shoulder flexion (°)	33	62.8 (23.9)	145.3 (13.9)	−82.5 (−90.7 to −74.2)	t(32) = −20.429	<0.001	<0.001	*d_z_* = −3.556
Craniovertebral angle (°)	33	25.7 (12.9)	11.2 (10.0)	14.5 (9.0 to 20.1)	t(32) = 5.355	<0.001	<0.001	*d_z_* = 0.932

SD = standard deviation; CI = confidence interval. Mean differences were calculated as pretest minus posttest; therefore, negative mean differences and negative Cohen’s *d_z_* values indicate higher posttest values, whereas positive values indicate lower posttest values. Holm-adjusted *p*-values control the familywise type I error rate across the 11 kinematic comparisons. Statistical conclusions were based on the adjusted *p*-values. All analyses were exploratory.

**Table 3:** Inter-rater reliability and measurement error for two-dimensional kinematic measurements.

Measurement	*n*	ICC(A,1)	95% CI	SEM (°)	MDC95 (°)
Ankle plantarflexion	33	0.985	0.959 to 0.998	0.72	1.99
Ankle dorsiflexion	33	0.534	−0.058 to 0.742	0.95	2.64
Knee flexion	33	0.998	0.993 to 0.999	0.88	2.44
Hip flexion	33	0.994	0.986 to 0.999	1.37	3.80
Shoulder flexion	33	0.996	0.988 to 1.000	1.47	4.07
Craniovertebral angle	33	0.978	0.953 to 0.995	1.86	5.14

Note. ICC(A,1) = two-way random-effects, absolute-agreement, single-measure intraclass correlation coefficient; CI = confidence interval; SEM = standard error of measurement; MDC95 = minimal detectable change at the 95% confidence level. Negative confidence limits were retained rather than truncated at zero.

**Table 4:** Intra-rater reliability of two-dimensional kinematic measurements (*n* = 33).

Outcome	*n*	ICC(A,1)	95% CI	SEM (°)	MDC_95_ (°)
Ankle plantarflexion	33	0.985	0.971–0.993	0.72	1.99
Ankle dorsiflexion	33	0.534	0.235–0.740	0.95	2.64
Knee flexion	33	0.999	0.997–0.999	0.73	2.02
Hip flexion	33	0.998	0.997–0.999	0.72	1.99
Shoulder flexion	33	1.000	0.999–1.000	0.51	1.41
Craniovertebral angle	33	0.998	0.995–0.999	0.56	1.56

Note. ICC(A,1) = two-way mixed-effects, absolute-agreement, single-measure intraclass correlation coefficient; CI = confidence interval; SEM = standard error of measurement; MDC_95_ = minimal detectable change at the 95% confidence level. SEM was calculated as SD_pooled_ × √(1 − ICC), and MDC_95_ was calculated as 1.96 × √2 × SEM. Interpretation followed the thresholds proposed by Koo and Li: <0.50, poor; 0.50–0.74, moderate; 0.75–0.89, good; and ≥0.90, excellent.

**Table 5:** Exploratory pretest-to-posttest differences in individual Hip Hinge Confidence Scale items.

Item	Pretest Median (IQR)	Posttest Median (IQR)	(W)	Unadjusted (*p*)	Holm-Adjusted (*p*)	Rank-Biserial Correlation
Confidence Q1 I am confident in performing a hip hinge to lift a lightweight object, such as a laundry basket or a small bag from the ground.	4 (4–5)	5 (4–5)	0.0	0.001	0.011	1.000
Confidence Q2 I am confident in using a hip hinge to lift a moderately heavy object, such as a grocery bag or small box.	5 (4–5)	5 (4–5)	12.0	0.013	0.059	0.736
Confidence Q3 I am confident in performing a hip hinge when moving furniture or heavier objects, like a chair or a box.	4 (3–5)	5 (4–5)	5.5	0.003	0.025	0.879
Confidence Q4 I am confident in performing a hip hinge when doing household tasks like vacuuming or sweeping.	4 (4–5)	5 (4–5)	20.5	0.017	0.059	0.658
Confidence Q5 I am confident in performing a hip hinge when squatting down to sit on or rise from a low chair.	4 (4–5)	5 (5–5)	5.5	0.001	0.012	0.908
Confidence Q6 I am confident in using a hip hinge when bending forward to stretch or tie my shoes.	4 (4–5)	5 (4–5)	4.0	0.013	0.059	0.855
Confidence Q7 I am confident in maintaining proper hip hinge form during lower-body exercises, such as deadlifts or kettlebell swings.	4 (3–5)	5 (4–5)	14.0	0.004	0.025	0.794
Confidence Q8 I am confident in using a hip hinge to prevent back strain when bending forward for extended periods, such as gardening or cleaning.	4 (3–5)	5 (4–5)	32.0	0.022	0.059	0.582
Confidence Q9 I am confident that I can perform a hip hinge repeatedly during physical activity without causing pain.	4 (3–5)	5 (4–5)	10.5	0.012	0.059	0.769
Confidence Q10 I am confident in keeping my back safe and avoiding pain while performing a hip hinge during any activity.	4 (3–5)	5 (4–5)	21.0	0.006	0.036	0.725

IQR = interquartile range. Individual items were rated from 1 (“not at all confident”) to 5 (“extremely confident”). Positive matched-pairs rank-biserial correlations indicate higher posttest responses. Holm-adjusted *p*-values control the familywise type I error rate across the 10 individual-item comparisons. Statistical conclusions were based on the adjusted *p*-values. All item-level analyses were exploratory.

## Data Availability

The data presented in this study are available on request from the corresponding author due to privacy and ethical restrictions related to the use of participant-level human subject data.

## Appendix A

**Table A1:** Hip Hinge Confidence Scale.

A hip hinge is a functional movement where you bend at the hips to safely lower your upper body. For each statement below, please check the number that best describes your level of confidence in performing a hip hinge safely and correctly during the listed activities.	**Not at all Confident-1**	**Slightly Confident-2**	**Somewhat Confident-3**	**Very Confident-4**	**Extremely Confident-5**
1. I am confident in performing a hip hinge to lift a lightweight object, such as a laundry basket or a small bag from the ground.					
2. I am confident in using a hip hinge to lift a moderately heavy object, such as a grocery bag or small box.					
3. I am confident in performing a hip hinge when moving furniture or heavier objects, like a chair or a box.					
4. I am confident in performing a hip hinge when doing household tasks like vacuuming or sweeping.					
5. I am confident in performing a hip hinge when squatting down to sit on or rise from a low chair.					
6. I am confident in using a hip hinge when bending forward to stretch or tie my shoes.					
7. I am confident in maintaining proper hip hinge form during lower-body exercises, such as deadlifts or kettlebell swings.					
8. I am confident in using a hip hinge to prevent back strain when bending forward for extended periods, such as gardening or cleaning.					
9. I am confident that I can perform a hip hinge repeatedly during physical activity without causing pain.					
10. I am confident in keeping my back safe and avoiding pain while performing a hip hinge during any activity.					

## Appendix B. Founder Exercise Instructions

The following standardized instructions were provided to participants using the scripted language presented below.

The exercise I will be teaching you today is called the Founder. The Founder Exercise is a tool for teaching you to hinge at the hips so that you can stabilize and strengthen your spine as your hips move and take on the load of your body.

1. Starting Position:
  ○ Step onto the mat and stand with your legs about shoulder-width apart or slightly wider.
  ○ Ensure your feet are parallel with a subtle degree of internal rotation (so both feet angle slightly inward).
2. Foot Position:
  ○ Maintain three points of contact with your feet (the heel, the base of the big toe, and the base of the little toe).
  ○ The weight should be in your heels so that the ends of your toes can spread.
3. Upper Body Position:
  ○ Lift your chest and eyes up.
  ○ Take a deep breath into the highest point of your nose, expanding your ribcage outward.
4. Head and Neck Position:
  ○ Chin up, chin tucked, chest up.
5. Movement:
  ○ With hands on hips, practice drawing the pelvis behind you 3 times.
  ○ Again, stand tall, lifting the chest in a deep breath towards that highest point in your nose.
  ○ Now we will add the arms to act as a counterbalance.
  ○ Anchor your hips and hands away from each other. As you hinge your hips back, your hands should move forward in your field of vision with your palms up. Keep the torso long and lifted. Allow your hands to counterbalance the movement at your hips.
  ○ Allow your knees to unlock so that they move with your hips. Engage your legs to pull inward (tensioning the feet towards the center).
  ○ Pull your hips and elbows as far away from each other as possible.
  ○ Pause and feel the load through your hamstrings, gluteal, and groin muscles. Now allow your knees to come forward slightly. Feel that the tension is shifted into the quadriceps on the front of your legs. This is not what we want. Lengthen the hamstrings on the backside of your legs, lifting the hips.
6. Common Mistakes to Avoid:
  ○ Squatting Instead of Hinging: Avoid bending your knees too much. Focus on pushing your hips back and unlocking the knees. The effort should be felt more in the backside of your body rather than your quads and knees.
  ○ Bowing Out: Ensure you hinge at your hips rather than bending forward. If you bow out, you might feel the weight shift towards the front of your toes, which can cause your heels to lift off the ground. Rather, pull your hips behind your heels to create tension up the backside of your legs.
